# Supplementary material for: Influence of APOE4 Genotypes on Nutrient–Cognition Relationship in Taiwanese Older Adults: Longitudinal Findings from the HALST
Source: Nutrients. 2025 Dec 28;18(1):106. doi: 10.3390/nu18010106 (PMC12788161; doi:10.3390/nu18010106)
Supplement: Supplementary file 1 [file nutrients-18-00106-s001.zip › nutrients-4022914-Supplementary data.pdf]

Supplementary data

Table S1 (Excel). Measurement information for questionnaire and biomarker variables.

Table S2. Egienvalues, proportion of variance, and cumulative of variance of Principal components (PC) 1 to 31.

PC	Eigenvalues	Proportion of variance	Cumulative of variance
PC1	18.875	0.609	0.609
PC2	4.017	0.130	0.738
PC3	1.417	0.046	0.784
PC4	1.130	0.036	0.821
PC5	1.006	0.032	0.853
PC6	0.766	0.025	0.878
PC7	0.621	0.020	0.898
PC8	0.548	0.018	0.916
PC9	0.503	0.016	0.932
PC10	0.388	0.013	0.944
PC11	0.353	0.011	0.956
PC12	0.283	0.009	0.965
PC13	0.201	0.006	0.971
PC14	0.171	0.006	0.977
PC15	0.131	0.004	0.981
PC16	0.127	0.004	0.985
PC17	0.114	0.004	0.989
PC18	0.089	0.003	0.992
PC19	0.070	0.002	0.994
PC20	0.049	0.002	0.995
PC21	0.034	0.001	0.997
PC22	0.029	0.001	0.998
PC23	0.018	0.001	0.998
PC24	0.015	0.000	0.999
PC25	0.012	0.000	0.999
PC26	0.009	0.000	0.999
PC27	0.007	0.000	1.000
PC28	0.007	0.000	1.000
PC29	0.004	0.000	1.000
PC30	0.004	0.000	1.000
PC31	0.001	0.000	1.000

Footnote: Proportion of variance = eigenvalues / sum(eigenvalues). Cumulative of variance = cumulative sum (proportion of variance).

Table S3. Associations of dietary transformed components (TC1 and TC2) with circulating nutrition- and lipid-related biomarkers.

Dependent Variable	Independent Variables	B	95% Confidence interval	p	FDR q
**Folate**	TC1	0.112	(0.039, 0.185)	**0.003**	**0.021**
**HDL**	TC1	-0.109	(-0.193, -0.025)	**0.011**	**0.039**
**Vitamin D**	TC1	-0.089	(-0.253, 0.075)	0.191	0.405
**Triglyceride**	TC1	0.544	(-0.379, 1.466)	0.248	0.405

**Vitamin B12**	TC1	-7.312	(-18.287, 3.663)	0.289	0.405
**LDL**	TC1	-0.031	(-0.320, 0.259)	0.835	0.881
**Cholesterol**	TC1	-0.026	(-0.363, 0.311)	0.881	0.881

**Dependent Variable**	**Independent Variables**	**B**	**95% Confidence interval**	**p**	**FDR q**
**LDL**	TC2	0.380	(0.311, 0.673)	**0.011**	**0.042**
**Cholesterol**	TC2	0.439	(0.097, 0.780)	**0.012**	**0.042**
**Folate**	TC2	0.044	(-0.030, 0.118)	0.247	0.464
**Vitamin B12**	TC2	-6.348	(-17.507, 4.811)	0.265	0.464
**HDL**	TC2	-0.022	(-0.107, 0.064)	0.623	0.752
**Triglyceride**	TC2	0.182	(-0.754, 1.119)	0.702	0.752
**Vitamin D**	TC2	0.027	(-0.139, 0.193)	0.752	0.752

Footnote: Linear regression analyses were used to examine relationships between the dietary change–derived transformed components (TC1, TC2) and blood biomarkers including triglycerides (TG), LDL-C, HDL-C, total cholesterol, vitamin D, vitamin B12, and folate. Shown are standardized beta coefficients, 95% confidence interval, nominal p values, and Benjamini–Hochberg false discovery rate–adjusted q values (FDR q).

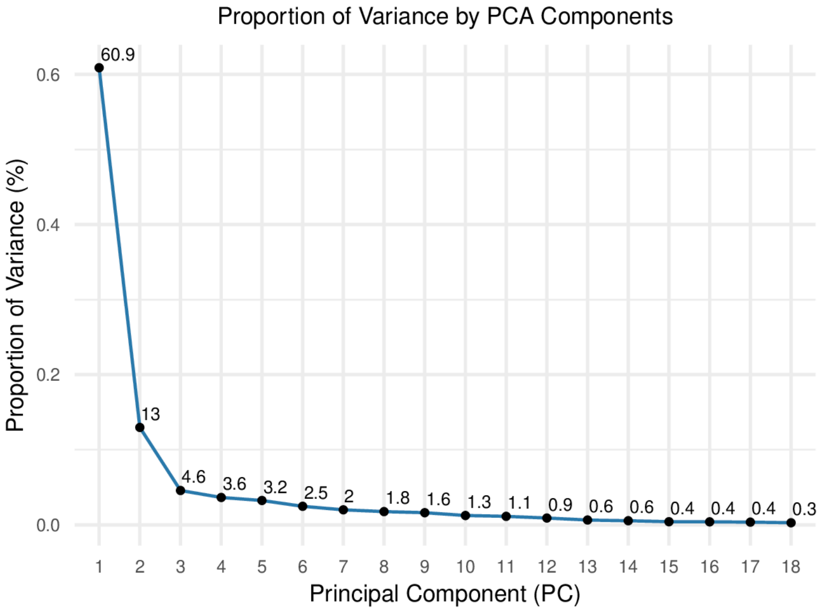

Figure S1. Proportion of variance explained by PCA components. Scree plot made by proportion variance (%) and PCA components. PC19-31 were not shown.
